# Machine Learning-Based Prediction Model of Pilgrims’ Tiredness During Hajj Using Smartwatch Physiological and Mobility Indicators

**DOI:** 10.3390/s26175625

**Published:** 2026-09-04

**Authors:** Mazin Alshamrani, Shema Alhazmi, Tarik Alafif, Thamir M. Qadah, Malak Aljabri, Walla Al-Eidarous, Abdullah Alhawsawi, Majed Farrash

**Affiliations:** 1 Department of Information and Scientific Services, Custodian of the Two Holy Mosques Institute for Hajj and Umrah Research, Umm Al-Qura University, Makkah 24381, Saudi Arabia; anhawsawi@uqu.edu.sa; 2 Applied College, Umm Al-Qura University, Makkah 24381, Saudi Arabia; smmalhazmi@uqu.edu.sa; 3 Department of Computer Science, Jamoum University College, Umm Al-Qura University, Makkah 24381, Saudi Arabia; tkafif@uqu.edu.sa; 4 Department of Computer and Network Engineering, College of Computing, Umm Al-Qura University, Makkah 24381, Saudi Arabia; tmqadah@uqu.edu.sa (T.M.Q.); mssjabri@uqu.edu.sa (M.A.); wmeidarous@uqu.edu.sa (W.A.-E.); 5 Department of Computer Science and Artificial Intelligence, College of Computing, Umm Al-Qura University, Makkah 24381, Saudi Arabia; mmfarrash@uqu.edu.sa

**Keywords:** Hajj, wearable sensing, tiredness prediction, physiological signals, mobility indicators, machine learning, synthetic data augmentation, AdaBoost, Decision Tree

## Abstract

Pilgrim safety during Hajj is challenged by dense crowds, sustained physical exertion, and demanding environmental conditions that can increase tiredness and compromise well-being. Although wearable sensing enables objective monitoring, the reviewed literature provides limited evidence on how synthetic augmentation affects predictive performance and cross-participant generalization when wearable data are scarce. To address this gap, this study presents an integrated framework that combines correlation- and domain-informed feature screening with statistically constrained synthetic data generation and a complementary assessment of predictive performance and synthetic data fidelity. Using the Hajj 1445 (June 2024) Apple Watch dataset comprising 120 records from three participants, the study compares classical resampling, univariate normal generation, multivariate normal (MVN) sampling, and covariance-aware generation using Cholesky decomposition and Mahalanobis distance filtering under a consistent classifier evaluation protocol. The highest observed record-level test accuracy is 93.33%, achieved by a Decision Tree using MVN sampling with moderate clipping (±1.4). Leave-one-participant-out evaluation of the same configuration yields a mean accuracy of 68.65% (SD = 19.72%), indicating lower cross-participant generalization. The regularized MVN–Cholesky–Mahalanobis configuration attains a composite synthetic-data quality score of 79.27%. These results show that statistically constrained synthetic augmentation can support tiredness prediction when real data are limited, while the participant-grouped results highlight the need for validation using larger and more diverse Hajj cohorts.

## 1. Introduction

Hajj is one of the largest mass gatherings in the world, where operational safety depends not only on logistics and crowd control, but also on the timely recognition of emerging health risks. During the pilgrimage, sustained walking, time-compressed movement between ritual sites, and prolonged exposure to demanding conditions can accelerate physical fatigue. When fatigue is not detected early, it can increase the probability of adverse events and reduce the ability of a pilgrim to complete activities safely. These realities motivate practical monitoring approaches that can operate in-field, with minimal disruption to pilgrims and without relying solely on post hoc reporting.

Wearable devices provide a direct mechanism for unobtrusive health monitoring during real-world activity. Smartwatches can capture physiological indicators and mobility-related signals that reflect short-term workload and recovery dynamics during movement. Compared with purely survey-based assessments, wearable sensing offers a higher temporal resolution view of the pilgrim’s state, potentially enabling fatigue-aware decision support. In our earlier published work [1], we introduced and analyzed a Hajj wearable dataset and demonstrated how combining physiological measurements with contextual descriptors can support structured interpretation of pilgrims’ stress and fatigue. The present paper follows that work by shifting from retrospective analysis to predictive models, that can infer tiredness levels from smartwatch-derived features.

A central challenge is that rigorous data collection during Hajj is inherently constrained. Real-world acquisition is affected by operational limits, participant availability, and the complexity of collecting and labeling health states in a dense, ethically sensitive environment. Consequently, the Hajj 1445 dataset used in this paper is low-resource by nature, comprising a limited number of records and a modest participant pool, while spanning multiple categories of information collected by Apple Watch devices. Training supervised models under these conditions requires careful handling of input features and a disciplined strategy to improve learning robustness without distorting the underlying data structure.

To address these constraints, this paper proposes a pipeline that emphasizes two principles. First, it prioritizes the most informative numerical attributes for tiredness prediction through correlation-driven feature screening. This step aims to concentrate model capacity on variables that are meaningfully associated with tiredness behavior, while de-prioritizing weakly related variables. Second, it investigates statistically grounded synthetic data augmentation techniques designed to expand the training distribution while preserving realistic multivariate dependencies between features. The proposed approach evaluates controlled sampling strategies, including multivariate normal (MVN) generation with covariance regularization and outlier filtering using Mahalanobis distance thresholds.

The work then benchmarks multiple machine learning classifiers under a consistent evaluation protocol and reports performance using standard classification metrics. In addition to predictive accuracy on held-out real data, the study evaluates synthetic-data utility using quality indicators that measure statistical similarity, class-wise alignment, and the extent to which models trained on synthetic samples can generalize back to the real dataset. This dual evaluation is important in low-resource health analytics: improvements in training size are only meaningful if the generated samples remain faithful to the real data distribution and do not inflate performance through unrealistic artifacts.

In summary, this paper contributes an integrated low-resource framework for predicting pilgrims’ tiredness levels during Hajj by combining feature selection, statistically constrained synthetic augmentation, and complementary evaluation of predictive performance and synthetic-data fidelity. Specifically, the paper

1. Integrates correlation- and domain-informed feature screening with smartwatch-based tiredness prediction using the Hajj 1445 dataset;
2. Systematically compares classical resampling, univariate generation, MVN sampling, covariance regularization, Cholesky decomposition, and Mahalanobis distance filtering under a consistent experimental protocol;
3. Jointly evaluates predictive performance and synthetic-data fidelity using classification, statistical-similarity, class-wise-similarity, and composite quality measures;
4. Complements the record-level experiments with leave-one-participant-out evaluation to assess cross-participant generalization under the limited participant pool.

The remainder of the paper is organized as follows. The next sections describe related work and the Hajj dataset, then present the preprocessing and modeling pipeline, followed by the experimental design and results. The paper concludes with a discussion of findings and practical implications for wearable-enabled monitoring in Hajj deployments.

## 2. Related Work

Stress and fatigue are common psychological and physiological responses in high-density crowds, especially under prolonged exposure, restricted mobility, excessive noise, and thermal discomfort. Research in crowd psychology indicates that overcrowding and reduced personal space increase stress levels, impair decision-making, and increase the likelihood of fatigue-related incidents [2,3]. In large-scale events and mass gatherings, such as religious pilgrimages and festivals, sustained physical exertion combined with cognitive overload further intensifies fatigue, likely causing unsafe behaviors and delayed reaction times [4]. Recent studies emphasize the need to monitor these states to improve safety management and facilitate timely interventions via data-driven intelligent systems [5].

Wearable health technologies utilize a variety of devices to continuously monitor physiological data and mitigate associated risks. Although smartwatches equipped with photoplethysmography (PPG) and electrocardiography (ECG) are widely used, ring-based and textile-based sensors have also been validated for stress detection [6,7,8,9]. Recent methodologies further improve these measurements by integrating biometric signals with environmental data, such as skin temperature and galvanic skin response (EDA), to more accurately assess autonomic nervous system (ANS) activity [10,11].

Effective interpretation of complex sensor data requires efficient algorithms. Consequently, machine learning (ML) techniques are widely used for detecting tiredness and stress because of relevance to health, safety, and performance. Early studies combined biometric signals (EEG, HRV, ECG, GSR) alongside classical ML classifiers such as Support Vector Machines (SVMs) and Random Forests to establish strong correlations [12,13]. More recently, advances in computer vision have enabled the application of deep learning methods for non-intrusive monitoring [14,15]. However, classical supervised ML remains widely used for structured healthcare data due to its interpretability.

Existing studies report competitive performance for tree-based ensemble algorithms in related healthcare tasks. For instance, research works by [16,17] demonstrate the operational advantages of Random Forest for medical diagnostic classification. Evaluation studies reinforce this perspective. The study in [18] reported that Random Forests achieved 97% accuracy in diabetes classification, outperforming SVMs and Logistic Regression. In the context of wearable analytics, [19] reported that Random Forest produced the lowest regression error (RMSE = 9.04 mg/dL) and the highest accuracy (78%) for predicting interstitial glucose. Similarly, [20] identified ensemble algorithms such as Gradient Boosting (99.05% accuracy) as leading approaches. Based on these observations, the present study adopts classical supervised ML.

In the context of Hajj and Umrah, ML capabilities are essential as technology evolves from basic digitization to continuous, context-aware monitoring. Applications in this setting operate as domain-constrained remote monitoring systems [21], which must address challenges such as the large number of pilgrims and device heterogeneity. Recent implementations illustrate this transition. For instance, ref. [1] described an Apple Watch workflow supported by mobile applications and web analytics that connects biometric indicators to geospatial descriptors, confirming that stress dynamics require a multidimensional approach. Complementary studies expand this domain; ref. [22] proposed an expert system integrating wearables and GPS to mitigate safety risks such as stampedes. In parallel, Hajj Bracelets have been explored for emergency alerts [23], while subjective satisfaction has been evaluated alongside physical load to contextualize well-being [1].

Despite these developments, significant challenges persist in effectively deploying such systems. Remote sensing in uncontrolled environments is restricted by insufficient data quality and quantity [24]. Data consistency is further affected by noise, participant compliance, and hardware limitations [25], while privacy regulations restrict access to labeled datasets. Although emerging techniques such as federated learning provide potential solutions for processing wearable data without compromising privacy [26], and adaptive algorithms can enable personalized responses [27], implementation remains complex. Collectively, these factors contribute to small or imbalanced datasets, which affect the reliability of predictions.

To address data imbalance, a common challenge in wearable analytics, standard solutions include oversampling and synthetic data generation. Techniques range from the Synthetic Minority Oversampling Technique (SMOTE) [28] and Adaptive Synthetic Sampling (ADASYN) [29] to advanced Generative Adversarial Networks (GANs) [30]. The Hajj 1445 dataset introduced in [1] provides a low-resource setting for evaluating these approaches. Structure-preserving statistical generation using covariance-aware sampling, Cholesky decomposition, and Mahalanobis distance filtering has been investigated in related domains [31,32,33]. However, the reviewed literature provides limited evidence on how synthetic augmentation affects both tiredness prediction and cross-participant generalization in participant-limited Hajj wearable data. This gap motivates the integrated evaluation conducted in this study.

## 3. Methodology

This research utilizes the Hajj 1445 dataset, which is designed to examine the heat stress levels of Hajj participants during the pilgrimage, as introduced in the study by [1]. The dataset comprises 120 records collected from three participants and includes 22 features representing personal, locational, and physiological health information extracted from their Apple Watch devices. A detailed description of all features is presented in Table 1.

The First Name and Last Name fields shown in Table 1 correspond to columns present in the original raw data collection; all participant identifiers were replaced with anonymized participant codes (P1–P3) prior to the analyses reported here, and no directly identifying information is included in the deposited dataset.

### 3.1. Data Preprocessing

**Figure 1:**
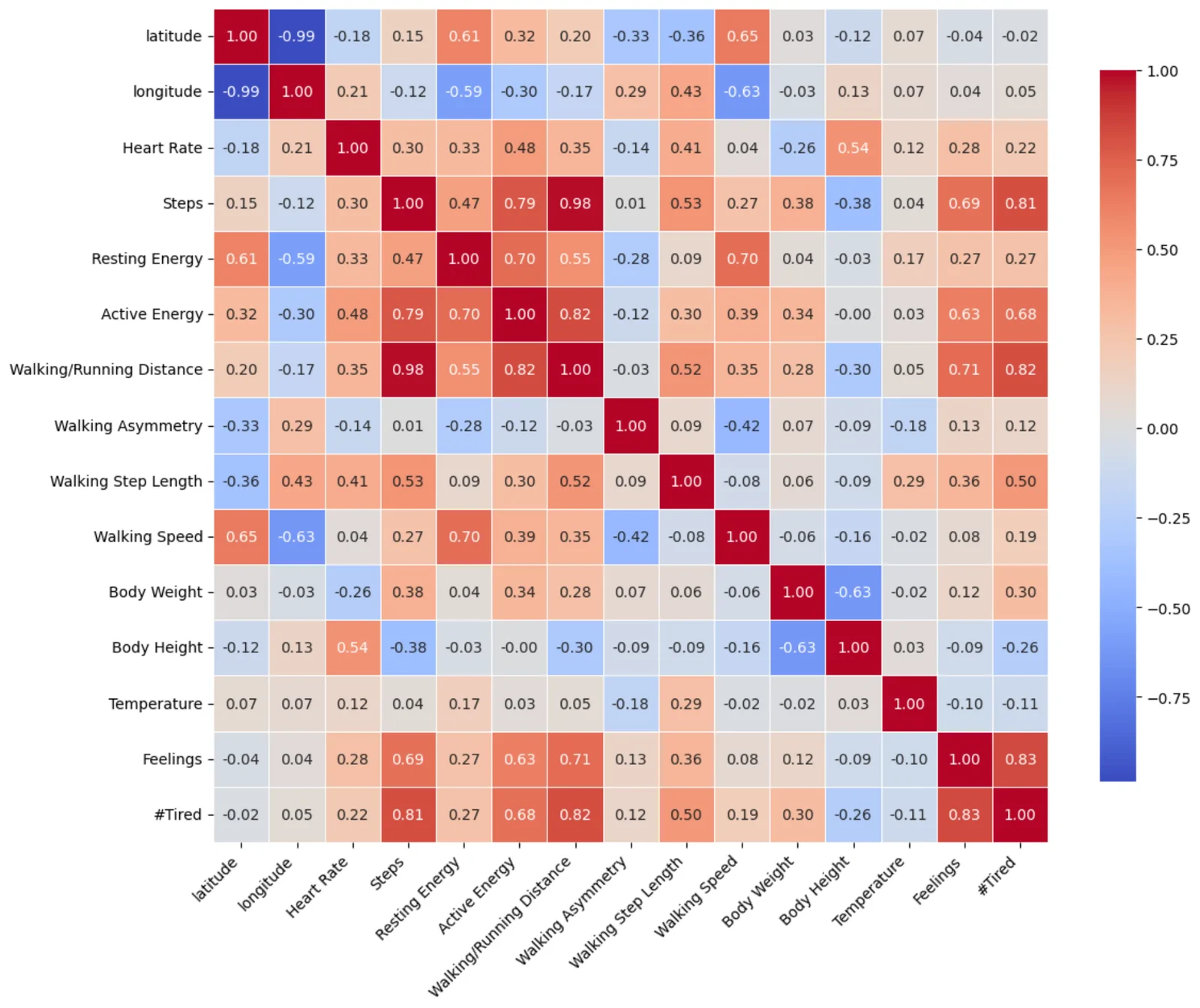
Feature selection using heatmap correlation analysis.

This research aims to analyze health-related information extracted from Apple Watch devices to characterize the behavior of pilgrims’ tiredness levels while performing Hajj rituals. Accordingly, we focus on numerical features and identify the most informative attributes that enhance prediction performance through correlation analysis using a heatmap. As illustrated in Figure 1, feature selection combined correlation strength with domain relevance. Latitude and Longitude were excluded due to negligible correlation with tiredness (|r|≤0.05) and because they describe measurement location rather than physiological state. Temperature was similarly excluded as a contextual/environmental descriptor. Body Height was excluded as a static anthropometric trait that does not vary with activity or fatigue during the pilgrimage, notwithstanding its moderate marginal correlation (r=−0.26). #Feelings was excluded despite its high correlation with #Tired (r=0.83) because both are self-reported subjective indicators collected via the same reporting mechanism, and including it as a predictor would risk label leakage because it is not independent of the self-reported target. Based on these criteria, the study selects nine numerical features representing physiological, mobility, and anthropometric information: Heart Rate, Steps, Resting Energy, Active Energy, Walking/Running Distance, Walking Asymmetry, Walking Step Length, Walking Speed, and Body Weight. Participant names have been replaced with anonymized codes in the deposited dataset; no directly identifying information is included. Body Weight was retained given its physiological relevance to exertion-related energy expenditure and mobility. As Body Weight takes only three participant-specific values in this dataset, Experiment V evaluates whether predictive performance persists when entire participants are held out.

In this research, several machine learning models are used to investigate the efficiency and effectiveness of training predictive models on a low-resource Hajj dataset.

Random Forest (RF) is an ensemble learning method constructed from multiple Classification and Regression Trees (CARTs). Each CART model estimates the conditional distribution of the target variable by recursively partitioning the predictor space into increasingly homogeneous regions through binary splits [34]. This process is represented as a tree structure, where each terminal node (leaf) corresponds to a subset of similar observations and encodes the outcome distribution for that group.

Random Forest models are particularly well suited for synthetic data applications, as they effectively capture complex, non-linear relationships that are difficult to model using traditional parametric approaches, while avoiding strong distributional assumptions. In addition, they can efficiently handle high-dimensional data and accommodate heterogeneous feature types. From a practical standpoint, Random Forests are computationally efficient, robust to overfitting, and require minimal hyperparameter tuning, making them easy to implement in applied machine learning settings [34].

Gradient Boosting (GB) is an ensemble learning approach that constructs a sequence of weak Decision Tree models, where each subsequent model iteratively improves performance by correcting the errors of its predecessors. The training process is guided by minimizing a predefined loss function through optimization along the negative gradient direction. This incremental learning strategy makes Gradient Boosting particularly well suited for data-constrained classification tasks, in which limited training samples may lead to instability and overfitting, and where class imbalance often biases models toward majority classes while overlooking rare but critical instances [35].

Decision Trees (DTs) are intuitive and widely used classification models that construct hierarchical, rule-based structures to map input features to target classes. By recursively partitioning the feature space into increasingly homogeneous subsets, DTs learn decision rules that are easy to interpret and directly reflect relationships within the data. Their non-parametric nature allows them to model complex and non-linear interactions without requiring prior assumptions about data distributions. Decision Trees can naturally handle numerical features, capture feature importance through split criteria, and provide transparent decision paths at the instance level, making them particularly suitable for exploratory analysis and small or heterogeneous datasets [36].

Support Vector Machines (SVMs) are supervised learning models grounded in the principle of structural risk minimization and Vapnik–Chervonenkis (VC) theory, and they are designed to achieve strong generalization by simultaneously maximizing the separating margin while controlling training errors. In the soft-margin formulation, SVM identifies an optimal hyperplane by minimizing a regularized objective function in which the user-defined penalty parameter (C) governs the trade-off between margin maximization and misclassification through slack variables. A key advantage of SVM is that its optimization problem is formulated as a convex quadratic programming task, which guarantees convergence to a global optimum and avoids entrapment in local minima [37].

k-Nearest Neighbors (KNN) is a simple, non-parametric, distance-based algorithm that classifies instances according to the majority label of their (k) nearest neighbors. Its lack of distributional assumptions allows it to model non-linear relationships through local neighborhood structures. With appropriate normalization and weighting, KNN provides a simple non-parametric baseline, although its performance can be sensitive to feature scaling, dimensionality, and class imbalance [38].

Logistic Regression (LR) is a classical linear, probabilistic classifier commonly used as a baseline due to its simplicity and interpretability. However, in data-constrained environments, LR often exhibits limited performance because it lacks inherent mechanisms to ensure stability when training samples are scarce. Its effectiveness further declines under class imbalance, where predictions tend to favor the majority class and fail to capture minority patterns. As a result, LR generally performs best when class distributions are balanced and sufficient data are available, which limits its suitability for low-resource and imbalanced classification settings [39].

Naïve Bayes (NB) is a family of probabilistic classifiers derived from Bayes’ theorem and built on the simplifying assumption that features are conditionally independent given the class label. Under this assumption, the presence of one feature is considered independent of others once the class is known. This simple yet effective modeling strategy makes NB particularly attractive in limited-data settings, as it requires minimal computational resources and can achieve competitive performance without complex model structures [40].

AdaBoost (Adaptive Boosting) is an ensemble method that sequentially combines multiple weak learners, typically shallow Decision Trees, into a single strong classifier. After each round, misclassified training samples receive higher weight so subsequent learners focus on harder cases, and the final prediction is a weighted vote across all weak learners. AdaBoost was included as an additional ensemble baseline to evaluate sequential boosting under the study’s small-sample, imbalanced setting. In this study, AdaBoost was implemented with n_estimators = 50, learning_rate = 1.0, and random_state = 42, consistent with the fixed seed used throughout this study.

### 3.2. Evaluation Metrics

In this study, we aim to assess the performance of the classification models in conjunction with the quality of the generated synthetic data. To evaluate model performance, we employ four standard classification metrics: accuracy, precision, recall, and F1-score. Accuracy measures the proportion of correctly classified samples relative to the total number of observations and is defined as

(1) $$\mathrm{Accuracy}=\frac{TP+TN}{TP+TN+FP+FN}.$$

Precision quantifies the ratio of correctly predicted positive samples to all predicted positive samples:

(2) $$\mathrm{Precision}=\frac{TP}{TP+FP}.$$

Recall, also known as sensitivity, measures the proportion of actual positive samples that are correctly identified by the model:

(3) $$\mathrm{Recall}=\frac{TP}{TP+FN}.$$

Finally, the F1-score provides a harmonic mean of precision and recall, offering a balanced measure particularly useful when the class distribution is imbalanced:

(4) $$F1\text{-}\mathrm{Score}=\frac{2\times \mathrm{Precision}\times \mathrm{Recall}}{\mathrm{Precision}+\mathrm{Recall}}.$$

Together, these metrics offer a comprehensive evaluation of the synthetic-data-trained classifier’s ability to generalize to real-world data by capturing correctness, robustness, and sensitivity across tiredness levels.

Furthermore, to evaluate the quality and practical usefulness of the generated synthetic data, three complementary measures are employed: (1) statistical similarity, (2) class-wise similarity, and (3) classifier-based generalization performance.

1. Feature-Level Statistical Similarity
  For each numerical feature *f*, the similarity between real and synthetic distributions is computed by comparing their empirical means and standard deviations:
  (5) $$S_{f}=\frac{1}{2}\left[1-\frac{|\mu_{f}^{\left(r\right)}-\mu_{f}^{\left(s\right)}|}{\left|\mu_{f}^{\left(r\right)}\right|+\epsilon }+1-\frac{|\sigma_{f}^{\left(r\right)}-\sigma_{f}^{\left(s\right)}|}{\left|\sigma_{f}^{\left(r\right)}\right|+\epsilon }\right],$$
  where $\mu_{f}^{\left(r\right)}$ and $\mu_{f}^{\left(s\right)}$ denote the real and synthetic means, $\sigma_{f}^{\left(r\right)}$ and $\sigma_{f}^{\left(s\right)}$ denote the corresponding standard deviations, and ϵ is a small constant to avoid division by zero.
  The overall statistical similarity is obtained by averaging across all *p* features:
  (6) $$S_{\mathrm{stat}}=\frac{1}{p}\sum_{f=1}^{p}S_{f}.$$
2. Class-Wise Similarity
  To assess alignment at the class level, we compute a similarity score based on aggregated class statistics:
  (7) $$S_{\mathrm{class}}=1-\frac{\left|\mu_{c}^{\left(r\right)}-\mu_{c}^{\left(s\right)}\right|+\left|\sigma_{c}^{\left(r\right)}-\sigma_{c}^{\left(s\right)}\right|}{\left|\mu_{c}^{\left(r\right)}\right|+\left|\sigma_{c}^{\left(r\right)}\right|+\epsilon }.$$
  where $\mu_{c}^{\left(r\right)}$ and $\sigma_{c}^{\left(r\right)}$ denote the mean and standard deviation of class *c* in the real dataset, $\mu_{c}^{\left(s\right)}$ and $\sigma_{c}^{\left(s\right)}$ denote the corresponding statistics in the synthetic dataset, and ϵ is a small positive constant introduced to prevent division by zero.
  This metric quantifies the relative deviation between real and synthetic class-level distributions, where values closer to 1 indicate stronger statistical alignment and better preservation of class-specific properties.
3. Classifier-Based Generalization Performance
  To evaluate predictive usefulness, a classifier trained exclusively on synthetic data is tested on the real dataset. The classification accuracy is computed as
  (8) $$\mathrm{Accuracy}=\frac{1}{N}\sum_{i=1}^{N}I\left({\hat{y}}_{i}=y_{i}\right),$$
  where I(·) denotes the indicator function, $y_{i}$ is the true label, and ${\hat{y}}_{i}$ is the predicted label.
  “RF Synthetic-to-Real Accuracy (%)” reports the classifier-based synthetic-data quality metric defined in Equation (8). A fixed Random Forest classifier is trained exclusively on synthetic data and evaluated using the real-data evaluation employed for this quality metric. “Best Classifier (Accuracy)” reports the highest held-out real-test accuracy among the eight classifiers evaluated in the prediction experiments. These columns represent different evaluation procedures and should not be directly compared as competing classifier results.
4. Overall Quality Score
  The final synthetic-data quality score is defined as the average of the three components:
  (9) $$Q_{\mathrm{overall}}=\frac{S_{\mathrm{stat}}+S_{\mathrm{class}}+\mathrm{Accuracy}}{3}.$$
  This composite metric provides a combined assessment of distributional fidelity and downstream predictive utility. Equal weighting of the three components was adopted for simplicity, transparency, and interpretability and to avoid introducing additional weighting hyperparameters into the proof-of-concept framework. A sensitivity analysis of alternative weighting schemes is left to future work.

To support reproducibility, a fixed random seed (42) was used for data splitting and for all stochastic preprocessing steps, including synthetic-data generation and resampling. Experiments were implemented in Python 3.12.13 using scikit-learn 1.6.1, imbalanced-learn 0.14.2, NumPy 2.0.2, and pandas 2.2.2. The source code is available from the corresponding author upon reasonable request.

## 4. Experiments and Results

**Figure 2:**
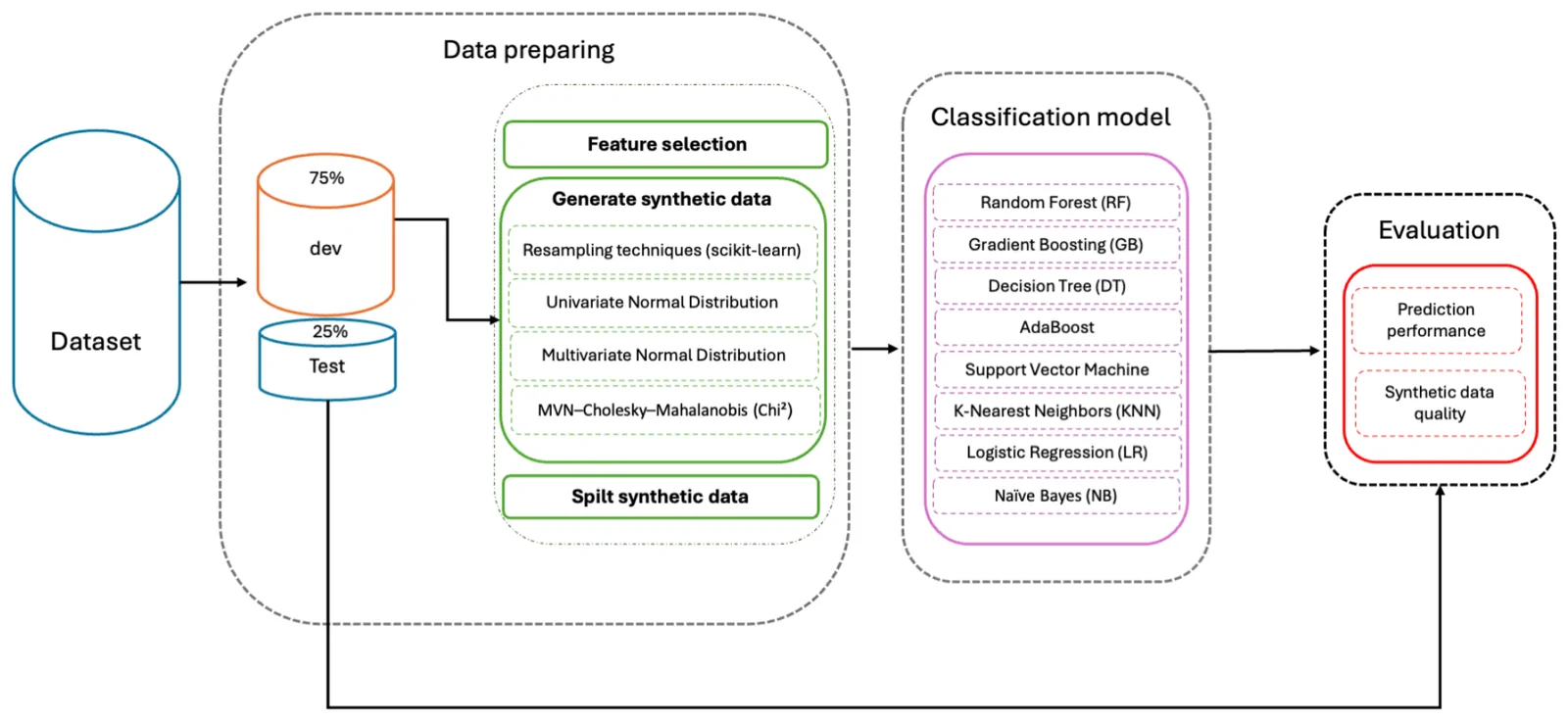
Proposed framework for synthetic data generation and tiredness prediction.

Figure 2 presents the overall experimental framework adopted in this study. The process begins with the original smartwatch-based dataset containing physiological and mobility-related features associated with different tiredness levels. During the data preparation stage, relevant features are selected and multiple synthetic data generation strategies are applied. These include classical resampling techniques (scikit-learn/imbalanced-learn), univariate normal distribution modeling, multivariate normal distribution (MVN) sampling, and MVN with Cholesky decomposition and Mahalanobis distance filtering (Chi-square constraint).

For the record-level experiments, class-conditional parameters are estimated exclusively from the training partition. Synthetic datasets are then generated at scales of 100, 200, 300, and 400 samples per class and used within the training and validation procedure. The held-out real test partition is excluded from parameter estimation and synthetic-data generation and is used only for final record-level evaluation.

The four clipping thresholds evaluated (±1.2, ±1.4, ±2.4, ±3.2 standard deviations) were selected via preliminary exploratory tuning to span a deliberately uneven range, from tight bounds that keep synthetic samples close to the class mean to permissive bounds approaching the tails of the estimated distribution, since the effect of a given increment on plausibility is non-linear near the center versus the tails of a Gaussian distribution.

The classification stage involves training multiple machine learning algorithms, including Random Forest, Gradient Boosting, Decision Tree, AdaBoost, Support Vector Machine, K-Nearest Neighbors, Logistic Regression, and Naïve Bayes.

Finally, the evaluation module assesses two complementary aspects: (1) prediction performance using accuracy, precision, recall, and F1-score on validation and real test sets, and (2) synthetic-data quality using statistical similarity, class-wise distribution alignment, classifier accuracy indicators, and an overall composite quality score. This structured framework enables a systematic comparison of augmentation strategies across different synthetic sample sizes and quantifies their impact on generalization performance under limited health data conditions.

### 4.1. Experiment I: Comparison with Imbalanced-Learn Resampling Methods

To evaluate the impact of different data resampling strategies on four-class fatigue classification under severe class imbalance, a series of controlled experiments is conducted using a stratified hold-out evaluation protocol. Numerical features are extracted following the feature-selection procedure described above and the target variable is defined as a four-class tiredness label. The dataset is partitioned into training and testing subsets using a stratified 75/25 split to preserve the original class distribution.

Each experiment follows a unified machine learning pipeline consisting of optional feature standardization, an imbalance-handling technique, and a classification model. In this study, the imbalanced-learn library is utilized to address class imbalance in the tiredness-level dataset. This library provides a comprehensive suite of resampling methods specifically designed for imbalanced learning problems and is directly applicable to our multi-class setting. Several resampling techniques are applied, including oversampling methods such as Random Oversampling (ROS) and SMOTE, undersampling methods such as Random Undersampling (RUS) and Tomek Links, as well as hybrid approaches including SMOTE–Tomek and SMOTE–ENN. These techniques are implemented within the training pipeline to rebalance the class distribution prior to model training, while preserving the original real dataset exclusively for evaluation. The objective is to systematically assess how traditional resampling strategies influence predictive performance in comparison with statistically generated synthetic data.

Oversampling-based methods are configured with a minimal neighborhood size to accommodate extremely small minority classes. For consistency, each classifier–resampling combination is trained on the same fixed training split and evaluated on a held-out test set using accuracy, F1-score, precision, and recall, ensuring a fair comparison across all classes. A stratified hold-out evaluation protocol is adopted to preserve the original class distribution and maintain stability in performance estimation under severe class imbalance. This experimental design enables a systematic and controlled assessment of classical resampling techniques under realistic, highly imbalanced conditions while ensuring reproducibility and reliable comparison across models.

**Figure 3:**
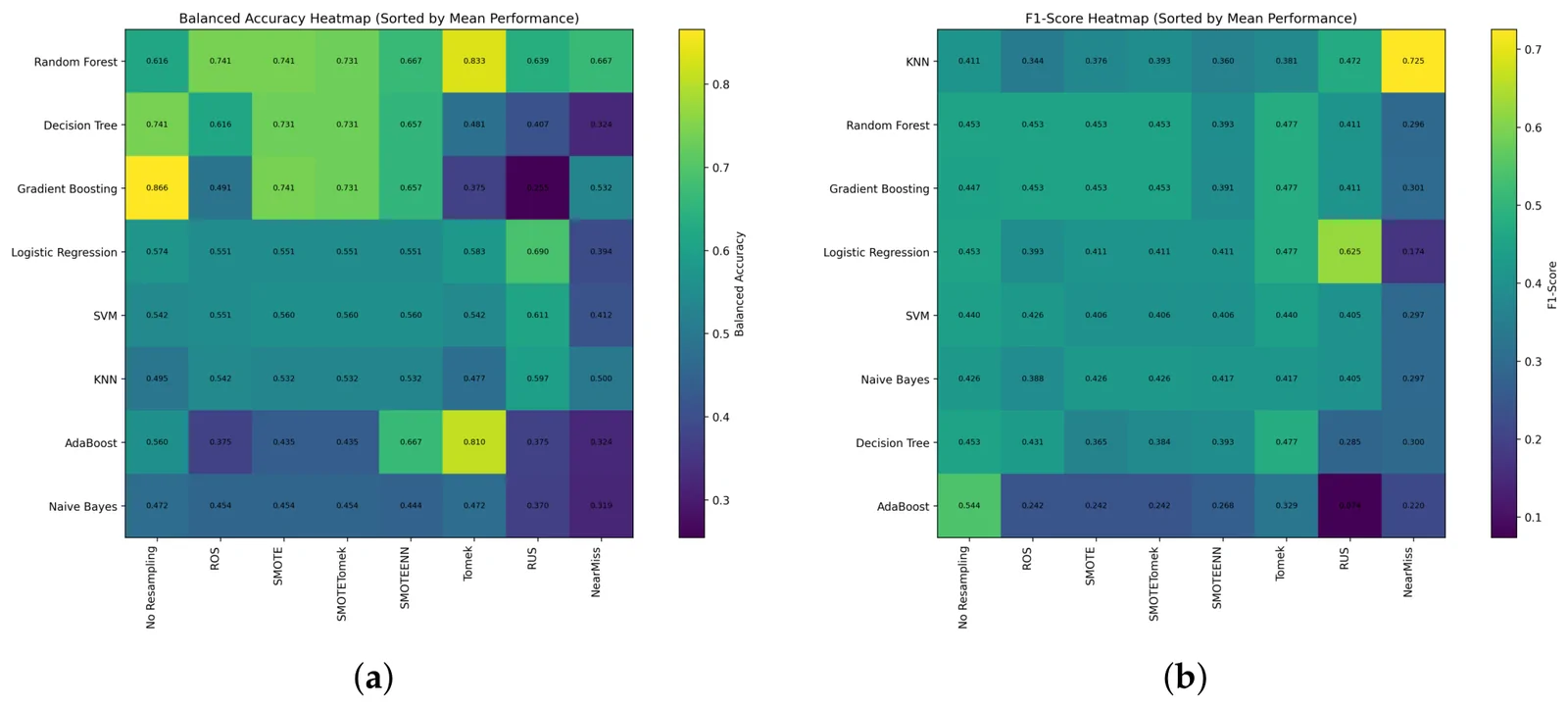
Performance comparison of classifiers under different resampling strategies. (**a**) Accuracy heatmap. (**b**) F1-score heatmap.

Figure 3a,b illustrate the heatmaps of accuracy and F1-score achieved by different classifiers under various resampling techniques. Overall, ensemble-based methods, particularly Random Forest and Gradient Boosting, demonstrate the most stable and consistent performance across most resampling scenarios. The highest accuracy (0.750) is achieved by KNN under NearMiss, with the corresponding highest F1-score of 0.725. However, KNN performance varies across the other resampling settings, so this result does not indicate consistent superiority. AdaBoost without resampling achieves an accuracy of 0.600, while several tree-based configurations remain around 0.48–0.50. Logistic Regression with RUS achieves the next highest F1-score of 0.625. Tomek Links produces modest changes for several tree-based classifiers, while Random Undersampling (RUS) and NearMiss reduce performance for many classifier configurations. Oversampling methods, including SMOTE and Random Oversampling, also do not consistently improve accuracy or F1-score across classifiers. Collectively, these results show that the effect of classical resampling is model-dependent in this four-class tiredness dataset, motivating the structured synthetic-data approaches evaluated in the subsequent experiments.

### 4.2. Experiment II: Univariate Normal Distribution (D1)

After evaluating classical resampling techniques, Experiment II introduces a probabilistic class-conditional generation approach to address their inconsistent and model-dependent performance under severe class imbalance while preserving statistical structure.

Table 2 consolidates the best-performing configuration under each clipping threshold by reporting statistical similarity, class-wise similarity, classifier accuracy, overall quality score, and the corresponding number of synthetic samples per class. Among all evaluated settings, the highest overall quality score (77.77%) is achieved with a clipping value of ±2.4. Under this configuration, AdaBoost achieves a real-test accuracy of 90.00% and an F1-score of 90.8% using 400 synthetic samples per class. Although tighter clipping (±1.2) yields slightly higher statistical similarity (57.02%), it does not provide the best overall quality. Conversely, moderate clipping values (±2.4 and ±3.2) improve class-wise similarity (above 92%) while maintaining stable RF synthetic-to-real accuracy (85.83%). These results suggest that moderate clipping offers a better balance between distributional alignment and predictive stability. Therefore, the configuration with clip = ±2.4 and 400 samples per class is selected for Experiment II because it yields the highest overall quality score (77.77%) while maintaining a real-test accuracy of 90.00%.

**Figure 4:**
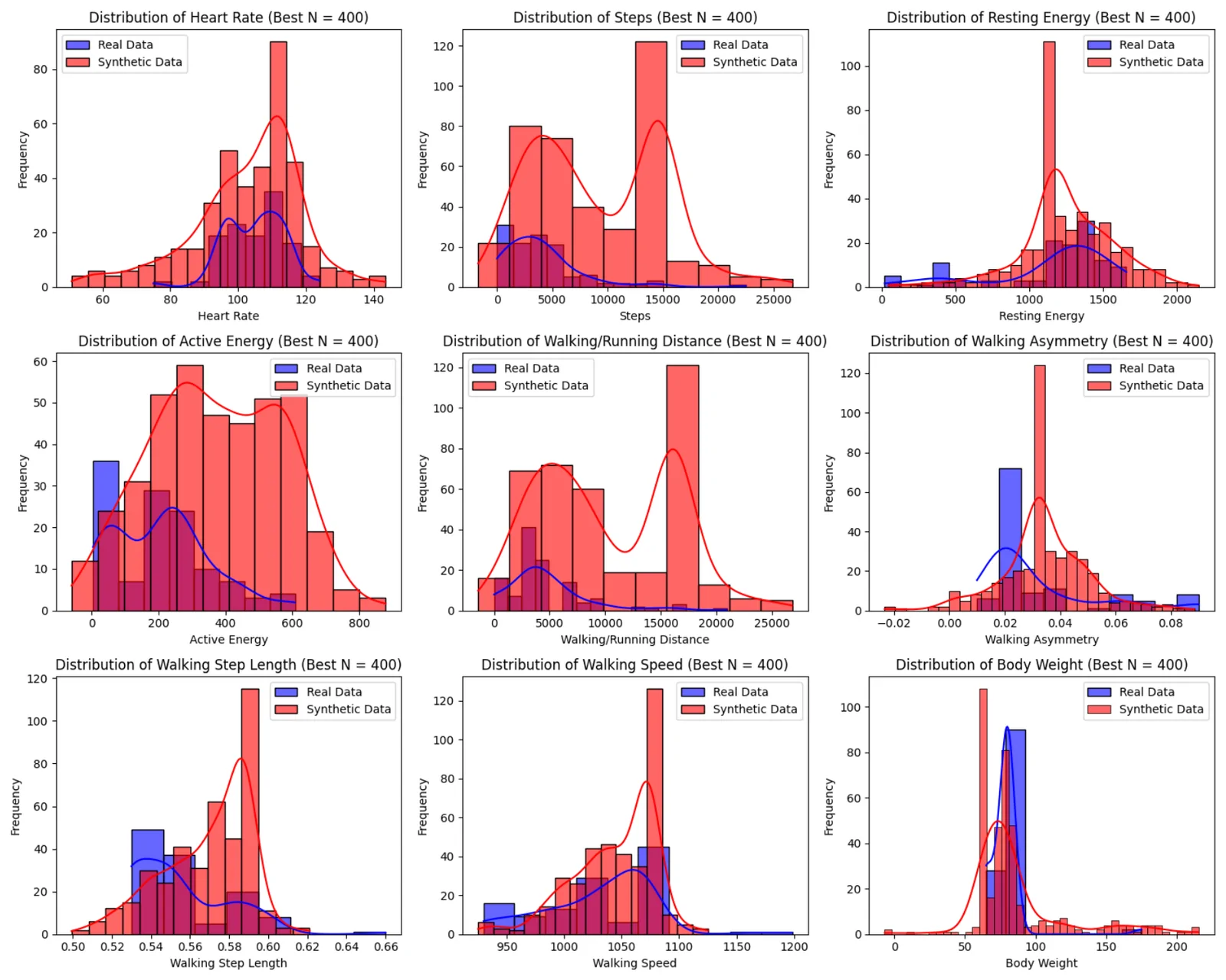
The best *N* based on real-data classification accuracy was obtained with N=400 samples per class at a clip value of 2.4, achieving (90%) accuracy using AdaBoost.

Figure 4 shows that, under the selected configuration of Experiment II (clip = ±2.4, *N* = 400) where *N* represents the number of synthetic samples generated per class, the synthetic data closely approximate the real feature distributions across most physiological and mobility-related variables. The central tendencies and overall dispersion patterns exhibit strong overlap, indicating that moderate clipping preserves the main structural characteristics of the data while maintaining statistical coherence. Although minor deviations appear in the tails for certain features, the controlled clipping effectively constrains extreme values and prevents unrealistic boundary expansion, thereby supporting stable downstream classification performance.

### 4.3. Experiment III: Multivariate Normal Distribution (MVN)

Although the univariate normal approach in Experiment II provided competitive performance, it assumes independence between features and does not preserve inter-feature correlations, which may distort the joint distribution despite matching marginal statistics. Therefore, Experiment III adopts a multivariate normal (MVN) framework to explicitly model the covariance structure and generate statistically coherent synthetic data.

In this experiment, synthetic samples are generated for all numerical features within each tiredness class using a multivariate normal distribution defined by the class-specific mean vector and covariance matrix. This formulation preserves inter-feature dependencies among physiological and mobility-related variables, allowing the synthetic data to better reflect the joint statistical structure of the original dataset. To prevent unrealistic boundary expansion, a controlled clipping mechanism is applied using predefined standard-deviation thresholds (±1.2, ±1.4, ±2.4, and ±3.2), while simultaneously constraining samples within observed class-specific feature ranges.

A balanced synthetic dataset is constructed by generating equal samples per tiredness category. Multiple machine learning classifiers are trained exclusively on synthetic data and evaluated on validation splits and real-world test data. Performance is measured using accuracy and F1-score, while synthetic-data quality is assessed through statistical similarity, class-wise similarity, and a composite overall quality score.

Table 3 summarizes the results across clipping thresholds. The highest overall quality score (78.90%) occurs at ±2.4, while the ±1.4 configuration yields the highest observed record-level test accuracy (93.33%) and F1-score (92.33%) using a Decision Tree with 400 synthetic samples per class. At ±1.4, statistical similarity is 57.17% and class-wise similarity is 89.77%, showing that the configuration with the highest predictive performance is not the configuration with the highest composite quality score.

Broader clipping thresholds increase class-wise similarity but do not improve the highest observed record-level accuracy. The configuration with clip = ±1.4 and 400 samples per class is selected as the record-level predictive setting because it yields the highest test accuracy (93.33%) and F1-score (92.33%), while the highest overall quality score (78.90%) occurs at ±2.4.

**Figure 5:**
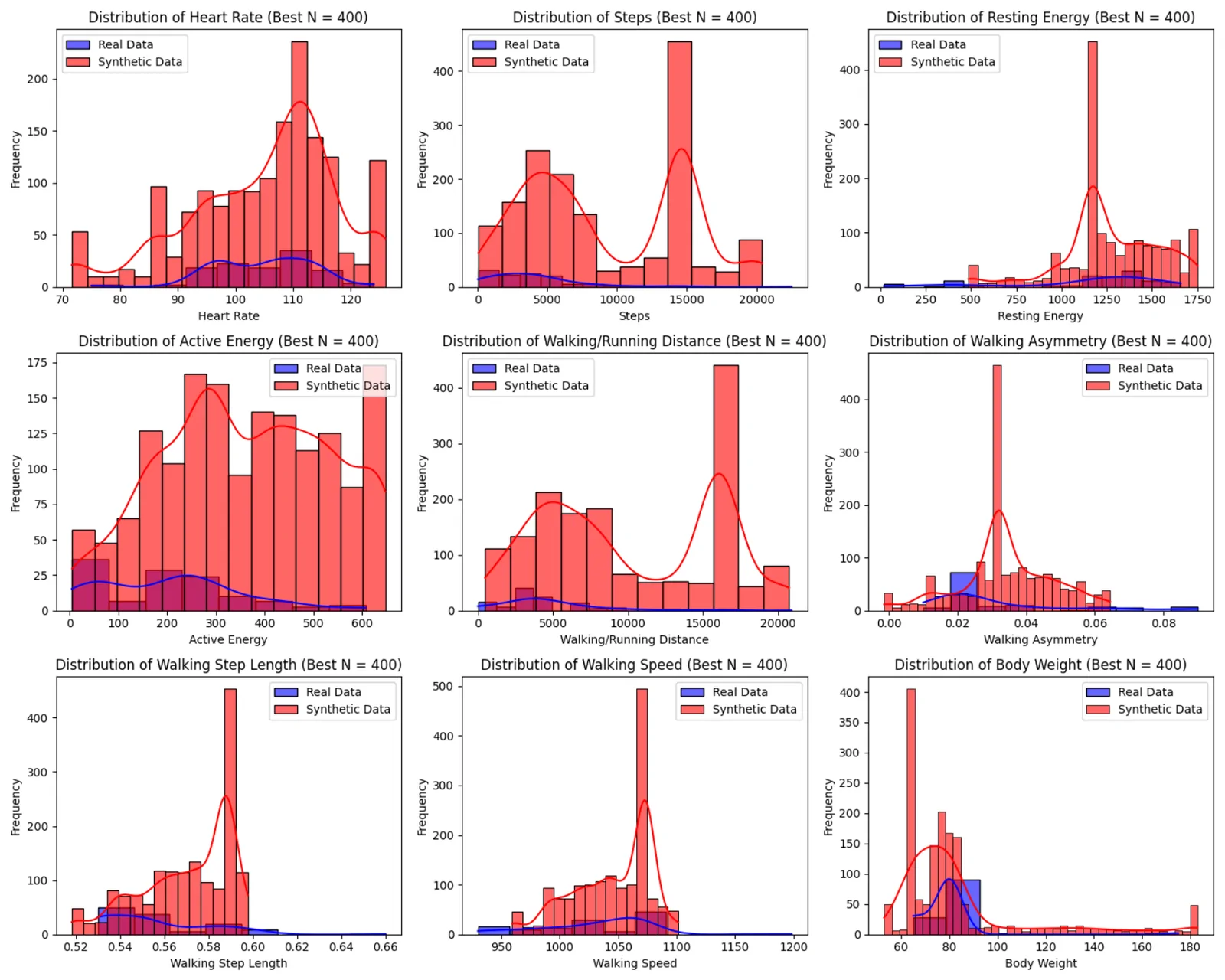
The best *N* based on real-data test accuracy was N=400 synthetic samples per class, achieving a test accuracy of (93%) using the Decision Tree classifier.

Figure 5 shows the distributional comparison between real and synthetic data under the selected record-level configuration of Experiment III (clip = ±1.4, (N = 400)). The synthetic samples closely align with the central tendencies and dispersion of most physiological and mobility features, indicating effective preservation of the covariance structure. Although slight deviations appear in the tails for certain skewed variables, the controlled clipping mechanism prevents unrealistic extremes, confirming strong distributional fidelity and supporting the selection of the ±1.4 setting.

Table 4 presents the performance and quality metrics obtained when synthetic samples are generated without applying clipping constraints. In this setting, the multivariate normal distribution is used directly with class-specific mean vectors and covariance matrices, while only enforcing observed feature ranges.

The results indicate that increasing the number of synthetic samples per class does not produce consistent improvements in predictive performance or overall quality. The highest overall quality score (80.25%) is achieved with 200 samples per class, where the classifier accuracy reaches 90.00%. However, the highest individual classifier accuracy (90.00%) is observed at both 300 samples (SVM) and 400 samples (Decision Tree), though these configurations yield slightly lower overall quality scores (79.45% and 79.22%, respectively). Statistical similarity remains relatively stable across sample sizes (approximately 58–59%), while class-wise similarity ranges between 89.08% and 91.67%.

Although the no-clipping configuration produces competitive predictive performance, it does not surpass the clipped MVN setting (±1.4), which achieves the higher peak record-level accuracy of 93.33%. The no-clipping distributions also show greater tail dispersion, while similarity and classification performance do not improve consistently across sample sizes. These results support the use of controlled clipping for the selected record-level configuration in Experiment III.

**Figure 6:**
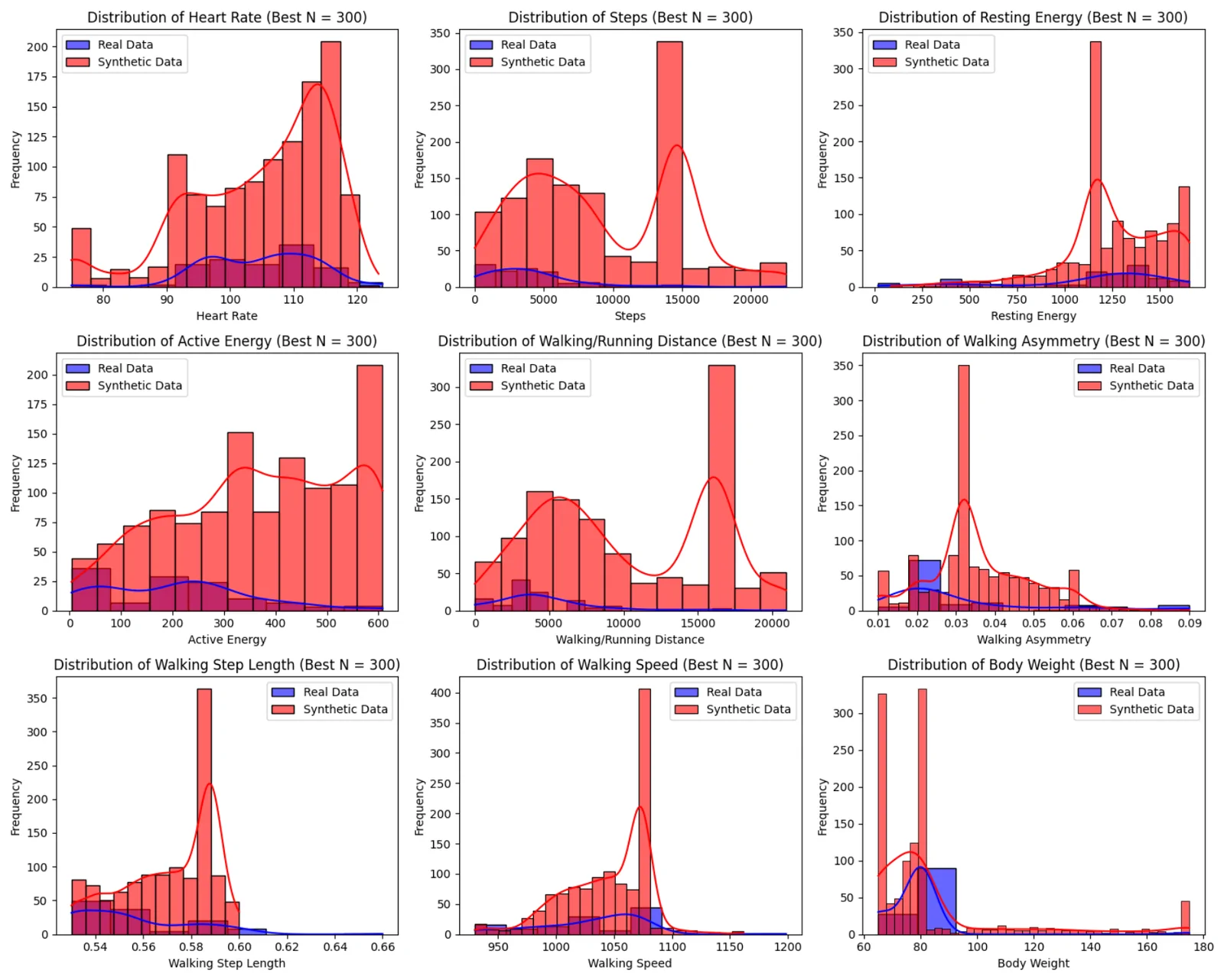
The best *N* based on real-data test accuracy was N=300 synthetic samples per class, achieving a test accuracy of (90%) using the SVM.

Figure 6 shows that, without clipping, the synthetic data align well with the real data in the central regions of most features, indicating that the multivariate normal model captures overall structure and correlations effectively. However, increased dispersion appears in the distribution tails—especially for energy-related variables and body weight—where synthetic samples exhibit heavier spread and occasional extremes. This greater boundary variability likely limits generalization performance, explaining why the no-clipping configuration does not outperform the moderately clipped setting.

### 4.4. Experiment IV: Multivariate Normal Distribution with Cholesky Decomposition and Mahalanobis Filtering (Chi-Square)

Although the MVN approach preserves inter-feature correlations, it relies mainly on covariance estimation and clipping, which control marginal ranges but do not ensure statistical validity in the full multivariate space. As a result, some synthetic samples may fall in low-probability or implausible regions of the joint distribution. To overcome this, Experiment IV introduces covariance regularization and Mahalanobis distance filtering, enforcing a chi-square confidence constraint to ensure probabilistic consistency and improve distributional fidelity and generalization performance.

In this experiment, synthetic data are generated for each tiredness category using a multivariate statistical modeling framework designed to preserve the joint structure among the real physiological and activity-based features. The procedure consists of four main stages, described below.

1. Multivariate Normal Sampling
  Each class is modeled using a multivariate normal distribution (MVN):
  (10) $$X_{c}∼N\left(\mu_{c},\Sigma_{c}\right),$$
  where $\mu_{c}$ denotes the mean vector of class *c*, and $\Sigma_{c}$ represents the corresponding covariance matrix that captures the inter-feature dependency structure within that class. Class-wise descriptive statistics including the empirical mean vector, covariance matrix, and range boundaries are computed using the numerical features. Synthetic samples are then drawn from the MVN for each class.
2. Regularization
  To ensure numerical stability and guarantee that each covariance matrix is positive definite, a small diagonal regularization term is added:
  (11) $$\Sigma_{c}^{\prime }=\Sigma_{c}+\lambda I.$$
  where λ denotes a small regularization constant introduced to stabilize covariance estimation, and *I* represents the identity matrix. This regularization enables performing a valid Cholesky decomposition in the next step.
3. Cholesky Decomposition
  The regularized covariance matrix is factorized using Cholesky decomposition as
  (12) $$\Sigma_{c}^{\prime }=L_{c}L_{c}^{⊤},$$
  where $L_{c}$ is a lower triangular matrix.
  Synthetic samples are then generated according to
  (13) $$x=\mu_{c}+L_{c}z,$$
  where z∼N(0,I) is a standard multivariate normal random vector. This transformation ensures that $x∼N(\mu_{c},\Sigma_{c}^{\prime })$, thereby preserving the covariance structure and inter-feature correlations observed in the real data.
4. Mahalanobis Distance Filtering
  To eliminate statistically implausible synthetic samples, each generated vector x is evaluated using the squared Mahalanobis distance defined as
  (14) $$D_{M}^{2}\left(x\right)={\left(x-\mu_{c}\right)}^{⊤}\begin{array}{c} {\left(\Sigma_{c}^{\prime }\right)}^{-1} \end{array}\left(x-\mu_{c}\right).$$
  Under the sampling model $x∼N(\mu_{c},\Sigma_{c}^{\prime })$, the squared Mahalanobis distance follows a chi-square distribution with *p* degrees of freedom:
  (15) $$D_{M}^{2}∼\chi_{p}^{2}.$$
  A generated sample is retained only if it satisfies the chi-square confidence constraint:
  (16) $$D_{M}^{2}\left(x\right)\leq \chi_{p,\alpha }^{2},$$
  where *p* denotes the number of features (dimensionality of the feature space), and $\chi_{p,\alpha }^{2}$ represents the upper α-quantile of the chi-square distribution (typically α=0.99). This filtering step removes extreme outliers while preserving statistically plausible samples within the multivariate confidence region.

The resulting synthetic dataset is merged with the real data and used to train multiple machine learning classifiers, including Random Forest, Gradient Boosting, SVM, KNN, Logistic Regression, Naïve Bayes, AdaBoost, and Decision Trees. Performance is evaluated on training, validation, and real-data test sets using accuracy and F1-score. Synthetic-data quality is further assessed through statistical similarity metrics, class-wise distribution alignment, and a composite overall quality score.

Table 5 summarizes the results across synthetic sample sizes under the MVN–Cholesky–Mahalanobis framework. The highest overall quality score (79.27%) and RF synthetic-to-real accuracy (88.33%) occur with 400 synthetic samples per class. The highest individual held-out test accuracy is 90.00%, achieved by AdaBoost with 100 samples per class. Statistical similarity remains relatively stable across sample sizes (55.59–56.98%), while class-wise similarity reaches its maximum of 92.83% at 300 samples per class. Thus, the 400-sample configuration provides the highest overall quality within Experiment IV, while the 100-sample configuration provides the highest observed individual test accuracy.

**Figure 7:**
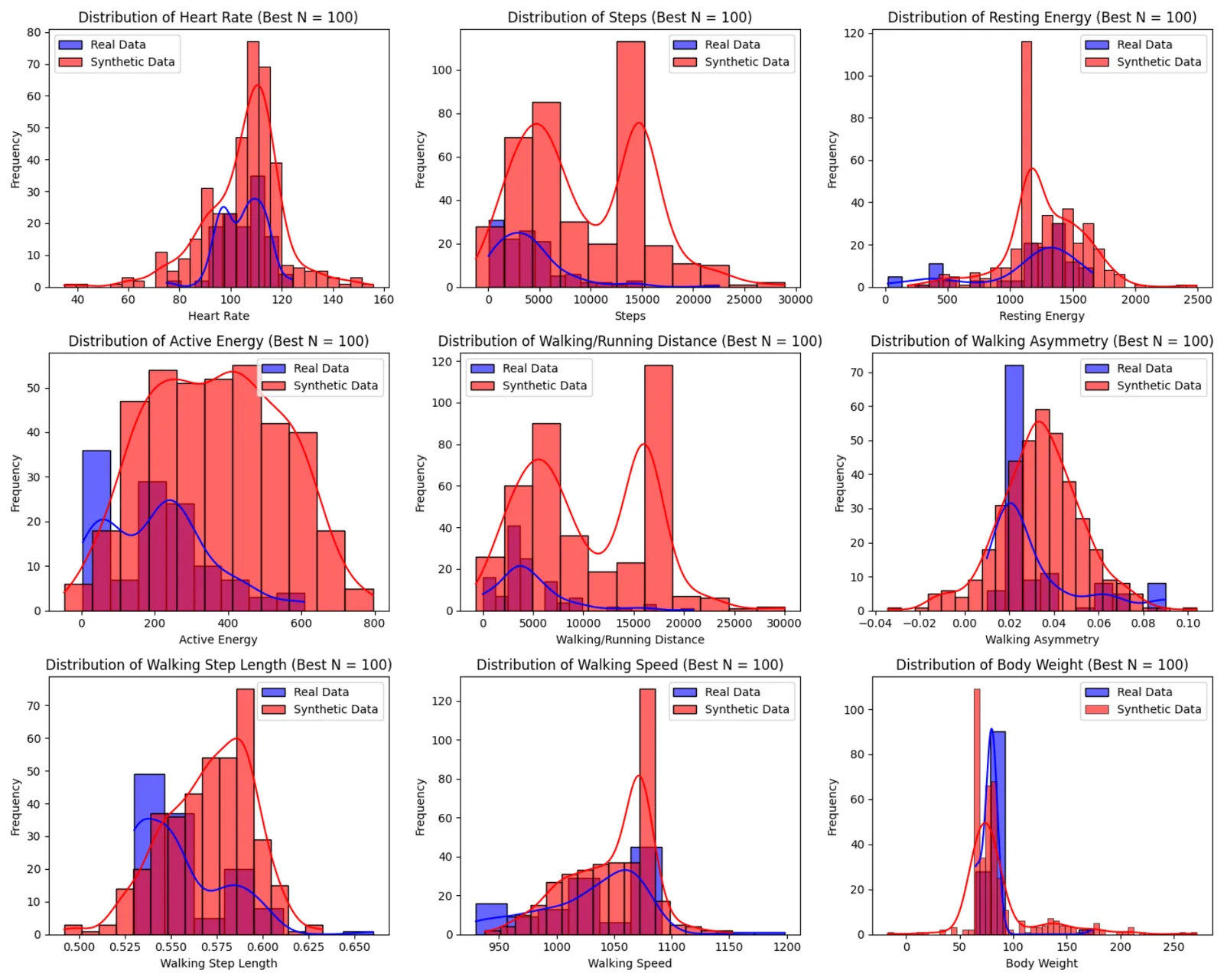
The best *N* based on real-data test accuracy was N=100 synthetic samples per class, achieving a test accuracy of (90.00%) using the AdaBoost classifier.

Figure 7 shows that the synthetic data generated using the structured MVN–Cholesky– Mahalanobis framework closely match the real distributions across all major features. The central regions are well aligned, and the tails are more controlled compared to earlier experiments, indicating effective outlier filtering and improved boundary regulation. This strong distributional consistency supports the high predictive performance reported in Table 5, confirming the stability and realism of the generated synthetic data.

### 4.5. Experiment V: Subject-Grouped Evaluation

Experiment II was additionally evaluated using a subject-grouped leave-one-participant-out GroupKFold procedure to complement the original record-level 75/25 split. The experiment employed the 1D Normal synthetic-data generation approach with an SVM classifier, a clipping range of ±2.4 standard deviations, and 400 synthetic samples per class. The dataset comprised three anonymized participants (P1, P2, and P3), contributing 90, 28, and 2 records, respectively. For each fold, synthetic data were generated exclusively from the training participants, while the held-out participant was completely excluded from synthetic-data generation and model training and was used only for evaluation. The SVM achieved fold-level accuracies of 57.78% for Wejdan, 89.29% for Hessa, and 0.00% for Ma. The resulting mean subject-grouped accuracy was 49.02% (SD = 45.28%). The substantial variation across folds indicates considerable inter-participant variability in model generalization. The zero accuracy for Ma should be interpreted cautiously because only two records were available for this participant, making this fold particularly sensitive to individual predictions.

Experiment III was similarly re-evaluated using a subject-grouped leave-one-participant-out GroupKFold procedure. In this experiment, synthetic data were generated using the multivariate normal distribution (MVN) approach, with a clipping range of ±1.4 standard deviations and 400 synthetic samples per class, followed by training of a Decision Tree classifier. For each fold, the MVN parameters were estimated exclusively from the training participants, and the held-out participant was excluded from both synthetic-data generation and model training. The Decision Tree achieved fold-level accuracies of 66.67% for P1, 89.29% for P2, and 50.00% for P3. The resulting mean subject-grouped accuracy was 68.65% (SD = 19.72%). As shown in Table 6, compared with Experiment II, the MVN-based Decision Tree demonstrated higher mean accuracy and substantially lower variability across participants. Nevertheless, the result for P3 should be interpreted with caution because this participant contributed only two test records, which limits the reliability of the corresponding fold-level estimate.

## 5. Discussion

This research investigates the feasibility and effectiveness of predicting pilgrims’ tiredness levels during Hajj using smartwatch-derived health data under low-resource conditions. Given the limited size of the Hajj 1445 dataset, comprising only 120 records collected from three participants, the study primarily addresses a key challenge in health analytics for large-scale events: how to build reliable ML models when real-world data collection is constrained. The experimental results provide important insights into feature relevance, model behavior, and the role of synthetic data generation in improving predictive performance.

The correlation-based feature selection process identifies associations between pilgrims’ tiredness levels and several physiological and mobility-related features extracted from Apple Watch devices. Heart rate, step count, energy expenditure, walking speed, and gait-related parameters show notable correlations with tiredness in this dataset. Latitude and Longitude were excluded because of negligible correlation (|r|≤0.05) and their locational role, while Temperature was excluded as a contextual/environmental descriptor. Body Height shows a moderate correlation (r=−0.26) but was excluded on domain grounds, as described in Section 3.1. These findings support the selected physiological, mobility, and anthropometric feature set used in the experiments.

Classifier performance varies across experiments and configurations, reflecting the difficulty of learning stable decision boundaries from the limited dataset. No single classifier is uniformly superior: AdaBoost, Decision Tree, and SVM achieve the highest observed accuracy under different synthetic-data configurations. These results support evaluating classifier choice jointly with the synthetic-data generation strategy.

The synthetic-data experiments provide deeper insights into predictive performance and distributional fidelity. The normal-distribution approach achieves competitive record-level performance under selected clipping thresholds, showing the importance of controlling synthetic boundary values. The MVN approach explicitly models inter-feature dependencies and achieves the highest observed record-level accuracy in Experiment III.

Notably, Experiment IV adds covariance regularization, Cholesky decomposition, and Mahalanobis-distance filtering to provide numerical stability and explicit control over multivariate plausibility. Within Experiment IV, the highest overall quality score is 79.27% with 400 synthetic samples per class, while the highest individual test accuracy is 90.00% with 100 samples per class. The added distributional control does not surpass the 93.33% peak record-level accuracy observed in Experiment III.

Peak record-level accuracy and synthetic-data quality capture different aspects of performance. Experiment III, using MVN with moderate clipping (±1.4), achieves the highest record-level test accuracy of 93.33%. Under leave-one-participant-out evaluation, the same configuration achieves a mean accuracy of 68.65% (SD = 19.72%), indicating lower cross-participant generalization. Experiment IV provides additional distributional control through covariance regularization and Mahalanobis filtering but does not exceed the peak record-level accuracy of Experiment III.

Despite the promising results, this study is limited by the small dataset of 120 records from three participants, which restricts generalizability across participants and broader Hajj populations. The record-level results, including the 93.33% peak accuracy, remain point estimates from a single stratified train–test split. The leave-one-participant-out evaluation provides a stricter participant-level assessment and shows lower accuracy with substantial between-participant variability. These grouped estimates also remain uncertain because only three participants are available, with P3 contributing only two records. No formal statistical significance test was performed between configurations; therefore, differences across clipping thresholds, sample sizes, and classifiers are interpreted descriptively. Larger and more participant-diverse datasets are required for stronger uncertainty estimation and external validation.

Overall, the experiments show that structured synthetic-data generation can improve record-level tiredness prediction in selected configurations, while the subject-grouped results indicate more limited participant-independent generalization. The findings therefore support the framework as a low-resource proof of concept and motivate evaluation using larger Hajj datasets.

## 6. Conclusions

This research presented an ML-based framework for predicting pilgrims’ tiredness levels during Hajj using smartwatch-derived physiological and mobility data under low-resource conditions. Leveraging the Hajj 1445 dataset, the study identified associations between tiredness and health indicators such as heart rate, energy expenditure, and gait-related features during pilgrimage activities. Given the limited availability of real-world data, the research explored multiple synthetic data generation strategies and evaluated their effects on predictive performance and synthetic-data fidelity.

The best-performing classifier varies across the evaluated configurations. Under the record-level evaluation, Experiment II selects AdaBoost with ±2.4 clipping and 400 synthetic samples per class, while Experiment III achieves the highest observed test accuracy of 93.33% using a Decision Tree with MVN generation and ±1.4 clipping. The same Experiment III configuration achieves a mean accuracy of 68.65% (SD = 19.72%) under leave-one-participant-out evaluation, showing lower participant-independent generalization. Experiment IV achieves its highest overall synthetic-data quality score of 79.27% through covariance regularization, Cholesky decomposition, and Mahalanobis filtering.

Overall, this research presents a low-resource proof-of-concept framework for tiredness prediction using wearable-derived features during Hajj. The findings provide a basis for broader participant-level validation and future development of wearable-enabled health-monitoring approaches for mass gatherings.

While the results are promising, several directions can be explored to extend and strengthen this research. First, future studies should incorporate larger and more diverse datasets collected from a broader population of pilgrims across multiple Hajj seasons to improve generalizability and reduce subject-specific bias. Second, integrating additional contextual factors—such as environmental conditions, crowd density, and ritual-specific activity patterns—could provide a more comprehensive understanding of fatigue dynamics.

From a methodological perspective, future work may explore advanced generative models, including variational autoencoders and Generative Adversarial Networks, to further enhance the realism and diversity of synthetic health data. Additionally, temporal modeling approaches such as recurrent neural networks or transformer-based architectures could be investigated to capture long-term fatigue progression over the course of the pilgrimage.

Following broader participant-level validation, integration with existing Hajj health and crowd-management platforms could support future real-time monitoring and automated alerts for medical and safety authorities. Edge-computing implementations and privacy-preserving learning techniques, such as federated learning, should also be considered to ensure scalability, data security, and compliance with ethical standards.

A web-based interface allowing new smartwatch measurements to be entered and tiredness predictions returned in real time would further bridge offline model development and field deployment.

In the long term, this research can be extended beyond Hajj to support health monitoring and safety management in other large-scale events and smart-city applications, contributing to Saudi Arabia’s vision of becoming a global leader in intelligent, safe, and sustainable mass-gathering management.

## Figures and Tables

**Table 1:** Description of Hajj 1445 dataset.

Feature	Description
First Name	The participant’s first name
Last Name	The participant’s last name
Gender	The gender of the participant
Location	The geographic place assigned to the participant during the Hajj journey
Stage	The specific Hajj ritual stage or activity phase (e.g., Mina, Arafat, Muzdalifah) during which the data were recorded
Latitude	The geographic latitude coordinate of the participant’s location at the time of measurement
Longitude	The geographic longitude coordinate of the participant’s location at the time of measurement
Heart Rate	The participant’s heart rate in beats per minute as measured by the smartwatch
Steps	The number of steps taken by the participant during a given time period
Resting Energy	The amount of energy (in calories) burned while the participant was at rest
Active Energy	The amount of energy (in calories) burned during physical activities
Walking/Running Distance	The total distance (in kilometers or miles) covered during walking or running activities
Walking Asymmetry	The asymmetry in the participant’s walking pattern, referring to an imbalance in movement,
	specifically differences between the left and right sides of the body during walking
Walking Step Length	The average length of the participant’s steps in centimeters
Walking Speed	The participant’s walking speed in kilometers per hour
Body Weight	The participant’s body weight in kilograms
Body Height	The participant’s body height in centimeters
Temperature	The ambient or body-related temperature recorded during data collection
Feelings	The self-reported emotional or physical state of the participant at a given time
Reason Of Feelings	The reported reason or contextual factor influencing the participant’s stated feelings
Tired	The reported tiredness level category (target variable)
Reason Of Tired	The participant’s stated reason explaining the reported tiredness level

**Table 2:** Performance of the Univariate Normal Distribution (D1) framework with clipping under different clipping thresholds (Experiment II). Abbreviations: LR, Logistic Regression; AB, AdaBoost.

Clip Value	Avg. Statistical Similarity (%)	Avg. Class-Wise Similarity (%)	RF Synthetic-to-Real Accuracy (%)	Overall Quality Score (%)	Best Classifier (Accuracy)	Best Classifier (F1-Score)	Samples per Class
1.2	57.02	89.67	85.00	77.23	LR (90.00%)	LR (90.8%)	200
1.4	56.22	90.52	85.83	77.52	LR (90.00%)	LR (90.8%)	400
2.4	54.80	92.68	85.83	77.77	AB (90.00%)	AB (90.8%)	400
3.2	54.69	92.77	85.83	77.76	LR (90.00%)	LR (90.8%)	300

**Table 3:** Performance of the multivariate normal distribution (MVN) framework with controlled clipping under different clipping thresholds. (Experiment III) Abbreviations: NB, Naïve Bayes; DT, Decision Tree; AB, AdaBoost.

Clip Value	Avg. Statistical Similarity (%)	Avg. Class-Wise Similarity (%)	Classifier Accuracy (%)	Overall Quality Score (%)	Best Classifier (Accuracy)	Best Classifier (F1-Score)	Samples per Class
1.2	58.83	89.69	87.50	78.67	NB (90.0%)	NB (88.7%)	300
1.4	57.17	89.77	88.33	78.43	DT (93.33%)	DT (92.33%)	400
2.4	55.73	92.64	88.33	78.90	AB (90.0%)	AB (88.85%)	300
3.2	55.23	92.56	86.67	78.15	NB (86.66%)	NB (86.00%)	200

**Table 4:** Performance of the multivariate normal distribution (MVN) framework without clipping under varying synthetic sample sizes (Experiment III). Abbreviations: GB, Gradient Boosting; SVM, Support Vector Machine; DT, Decision Tree.

Samples per Class	Avg. Statistical Similarity (%)	Avg. Class-Wise Similarity (%)	Classifier Accuracy (%)	Overall Quality Score (%)	Best Classifier (Accuracy)	Best Classifier (F1-Score)
100	59.15	89.08	85.00	77.74	GB (80.00%)	GB (80.47%)
200	59.09	91.67	90.00	80.25	SVM (86.66%)	SVM (84.37%)
300	59.27	91.58	87.50	79.45	SVM (90.00%)	SVM (87.22%)
400	58.25	90.24	89.17	79.22	DT (90.00%)	DT (89.29%)

**Table 5:** Performance of the MVN–Cholesky–Mahalanobis ($\chi^{2}$) framework under varying synthetic sample sizes (Experiment IV). Abbreviations: AB, AdaBoost; SVM, Support Vector Machine.

Samples per Class	Total Synthetic Samples	Avg. Statistical Similarity (%)	Avg. Class-Wise Similarity (%)	Classifier Accuracy (%)	Overall Quality Score (%)	Best Classifier (Accuracy)	Best Classifier (F1-Score)
100	388	55.59	91.46	86.67	77.91	AB (90.00%)	AB (89.29%)
200	777	55.61	91.80	87.50	78.30	SVM (83.33%)	SVM (83.22%)
300	1164	56.09	92.83	87.50	78.81	SVM (83.33%)	SVM (83.22%)
400	1552	56.98	92.51	88.33	79.27	AB (86.66%)	AB (86.00%)

**Table 6:** Subject-grouped evaluation results for Experiments II and III. Abbreviations: SVM, Support Vector Machine; MVN, multivariate normal distribution; SD, standard deviation.

Experiment	Synthetic Distribution	Classifier	Clip	Samples/Class	P1	P2	P3	Mean Accuracy	SD
Experiment II	1D Normal	SVM	±2.4	400	57.78%	89.29%	0.00%	49.02%	45.28%
Experiment III	MVN	Decision Tree	±1.4	400	66.67%	89.29%	50.00%	68.65%	19.72%

## Data Availability

The data presented in this study are available from the corresponding author upon reasonable request, due to participant privacy given the small number of subjects involved.
